# Digital entrepreneurship from cellular data: How omics afford the emergence of a new wave of digital ventures in health

**DOI:** 10.1007/s12525-023-00669-w

**Published:** 2023-09-16

**Authors:** Hannes Rothe, Katharina Barbara Lauer, Callum Talbot-Cooper, Daniel Juan Sivizaca Conde

**Affiliations:** 1 University of Duisburg Essen, Institute for Computer Science and Business Information Systems, Essen, Germany; 2 Airfinity Ltd., 71-75 Shelton St, London, WC2H 9JQ UK; 3 Department of Pathology, University of Cambridge, Cambridge, England; 4 Department of Information Systems, Freie Universität Berlin, Berlin, Germany

**Keywords:** Digital entrepreneurship, Digital innovation, Omics, Health data, External enabler, M150

## Abstract

Data has become an indispensable input, throughput, and output for the healthcare industry. In recent years, omics technologies such as genomics and proteomics have generated vast amounts of new data at the cellular level including molecular, structural, and functional levels. Cellular data holds the potential to innovate therapeutics, vaccines, diagnostics, consumer products, or even ancestry services. However, data at the cellular level is generated with rapidly evolving omics technologies. These technologies use scientific knowledge from resource-rich environments. This raises the question of how new ventures can use cellular-level data from omics technologies to create new products and scale their business. We report on a series of interviews and a focus group discussion with entrepreneurs, investors, and data providers. By conceptualizing omics technologies as external enablers, we show how characteristics of cellular-level data negatively affect the combination mechanisms that drive venture creation and growth. We illustrate how data characteristics set boundary conditions for innovation and entrepreneurship and highlight how ventures seek to mitigate their impact.

**Supplementary Information:**

The online version contains supplementary material available at 10.1007/s12525-023-00669-w.

## Introduction

Digital transformation has changed professional roles (Dougherty & Dunne, 2012), practices (Reckwitz 2002, Wessel et al., 2019), and institutions (Burton-Jones et al., 2020) in health and life sciences, leaving a deep mark on the sector over the past decade. Omics technologies, such as DNA sequencing, are collecting and analyzing data at the micro level with greater precision, speed, and scale, providing new insights into human, plant, animal, or microbiome life. Much of this data is called omics data, representing biological processes at the molecular level, including genomic (DNA), transcriptomic (RNA), proteomic (protein) and metabolomic (small molecule) data. Cellular-level data such as omics data are considered essential for digital innovation (Kulathinal et al., 2020) and have attracted the attention of information systems scholars interested in business model innovation (e.g., Thiebes et al., 2020), product innovation (Pentland et al., 2022), and governance (e.g., Jarvenpaa & Markus, 2018; Vassilakopoulou et al., 2019). Omics data have grown exponentially (Birney et al., 2017) since the culmination of the Human Genome Project in 2003 (National Human Genome Research Institute, 2015). Today, the tracking of RNA data of the SARS-CoV-2 virus is critical for vaccine production, diagnostic testing, and pandemic response policy. Omics data are also important for producing predictive medicine therapies for cancer (Chen & Snyder, 2013), helping to track and increase crop diversity in agriculture (Breed et al. 2019), or informing consumer goods companies where and how to improve toothpastes (Adams et al., 2020), clothing, creams, or lotions. This is done by integrating and analyzing omics data from different sources, often using sophisticated algorithms that simulate biological functions, cells, or even whole organisms (e.g. Cai et al., 2018; Karr et al., 2012). Omics data are therefore an important input, throughput, and output for digital innovation processes in a wide range of health applications.

The important role of data for innovation or entrepreneurship is not new to information systems researchers. However, omics data may reveal new boundary conditions for existing knowledge. In the literature on digital innovation, the role of digital technologies for innovation has mainly been addressed by focusing on the role of tools and features, e.g., reprogrammability (Langlois, 2002; Yoo et al., 2010) and replicability (Benkler, 2006; Henfridsson et al., 2014). Data is seen as an important element in the production of such tools and considered a “homogeneous” representation of our physical reality (Yoo et al., 2010). It can easily be used to quickly create or modify marketable offerings because it can be easily transferred, combined, and reused (Alaimo et al., 2020). Scholars of digital entrepreneurship build on these merits (Nambisan et al., 2017; von Briel et al., 2021) when they explain how data enables the creation and growth of entrepreneurial ventures (Nambisan, 2017; von Briel et al., 2018), that is, young and growth-oriented firms (Moreno & Casillas, 2007; Siegel et al., 1993). While data is considered homogeneous, the creation and growth of ventures is more influenced by the specificity and lack of interoperability of tools (von Briel et al., 2018). However, data has become critical to the value creation of digital ventures (Abbasi et al., 2016), especially as more companies produce machine learning-based products and services that rely heavily on data (Iansiti & Lakhani, 2020; Schulte-Althoff et al., 2020). Empirical studies suggest that data has an impact on the creation and growth of ventures because it enables ventures to adapt more quickly to market changes and ultimately create superior customer value (Gregory et al., 2020; Huang et al., 2017). These studies largely focus on highly structured and standardized transaction data, which is a predefined set of dimensions describing transactions between suppliers, customers, or users, including their recency, frequency, or value (Martens et al., 2016), in domains such as e-commerce (Huang et al., 2017), advertising (Aaltonen et al., 2021), or dating (Davidson & Vaast, 2010). Transactional data are mostly confined to the boundaries of a single company or platform, where the data are structured according to the standards of a single or few actors who can transfer, combine, and reuse the data with low transaction costs.

However, omics technologies generate data in an environment that is rich in resources—with expensive equipment, well-trained personnel, and highly regulated often personal medical information (e.g., from clinical trials). In most cases, therefore, omics data are generated by life scientists for the purpose of answering scientific hypotheses, i.e., the generation of insights into a phenomenon and the sharing of these insights with the public. However, because of its scientific origins, omics data is focused on specific research questions and methods and is often limited to a single and very specific use case. As omics technologies continue to advance, these data, their associated metadata, and policies are constantly evolving. Initiatives, such as the Global Alliance for Genomics and Health (https://www.ga4gh.org/), have introduced a first set of standards that still require prove of utility in research and clinical application for wide-spread use (Page et al., 2023; Rehm et al., 2021). As a result, standards for the description and categorization of micro-level entities are not widely adopted or take a long time to be used by the scientific community, leaving omics data in a constant state of flux (Powell, 2021). These characteristics may remain in contrast to the assumed inherent homogeneity of the data and its ability to enable entrepreneurship (von Briel et al., 2018). For this reason, we conducted interviews with entrepreneurs who produce products and services with omics data and invited investors and providers of data infrastructure to a focus group discussion in order to explore the following question: *How do characteristics of cellular-level data affect the creation and growth of digital ventures?*

Our qualitative study involved several rounds of interviews and a focus group discussion with entrepreneurs using omics technologies, investors, and data infrastructure providers. Some of our conversations were published as a podcast series (Data for Life, https://www.podomatic.com/podcasts/dataforlife.). In the following study, we outline how the specifics of omics data affect entrepreneurship. We conceptualize omics data as external enablers for digital ventures who combine these digital resources to start and grow a business (Davidsson et al., 2020; Davidsson et al., 2017; von Briel et al., 2018). Our study sheds new light on how omics data are high-dimensional, non-standardized, highly regulated, and have low reproducibility, and derive four propositions in how these effect the combination mechanism, i.e., access and reuse of digital resources. We find that both activities are individually affected by data characteristics and learn about interrelationships between both. To mitigate the negative impact of data characteristics on the combination mechanism, ventures require significant investment into other resources. We suggest new avenues for research in digital innovation and digital entrepreneurship, as we propose a contextualized combination mechanism for cellular-level data. Finally, we discuss how ventures become actors engage in making data sustainable through repurposing. We believe this research is important, particularly in light of the growing number of open data initiatives on cellular data, and large-scale regulations to open up health data for secondary analysis, such as the European Health Data Spaces.

## Background

### Data as external enablers for venturing

The homogenization of data, i.e., decoupling content from its original form (Yoo et al., 2010), lies at the core of digital innovation. Homogeneous data that has been created for one purpose can be exchanged, adapted, and eventually applied to yet another purpose at low marginal costs (Faulkner & Runde, 2019; Kallinikos et al., 2013). In advertising, firms combine a variety of data on user behavior to produce more personalized advertisements (Aaltonen et al., 2021; Alaimo, 2022). In healthcare, combining location and user data allows tracking patients and providing advice to caregivers (Wessel et al., 2019) and adding electronic health records supports clinicians making decisions on treatments in chronic diseases (Bardhan et al., 2020) or treatment planning in hospitals (Hansen & Baroody, 2020; Kohli & Tan, 2016).

Building on that line of argument, digital entrepreneurship research provides insights into how digital technologies such as digital data and functions enable entrepreneurial actions, i.e., venture creation and growth (von Briel et al., 2021). Conceptual work in that domain has found that characteristics of a digital technology, particularly their specificity and interoperability of algorithms and functions have an impact on the pace with which ventures are formed and how quickly they grow (von Briel et al., 2018). First empirical work on ventures working with transaction data suggests that the ability of a venture to swiftly adapt and release technology that utilizes such data has positive impacts on venture growth because ventures can use that data to improve their offerings (Huang et al., 2017). Digital technologies that help ventures utilize data (Davidsson et al., 2020) are considered external enablers in the venture creation process (Davidsson et al., 2017; von Briel et al., 2018). An external enabler is considered a “distinct, external circumstance [… that holds] the potential of playing an essential role in eliciting and/or enabling a variety of entrepreneurial endeavors” (Davidsson, 2015). The theory on external enablers explains how resource characteristics shape ventures, their offerings, or the process of venturing by explaining different mechanisms that specify cause-effect relationships. Since decoupling of content and form is a key element of digital technologies (Yoo et al., 2010), the *combination mechanism* (von Briel et al., 2018) is of particular importance for digital entrepreneurship. This mechanism explains the effect of multiple digital resources being used together for venturing. It not only takes individual characteristics of digital resources into account when studying their effects on venturing, but also considers interaction effects between these resources when successfully combined. Von Briel et al. (2018) lay out how the ability of hardware ventures to grow depends on specificity and interoperability of portable devices. Conditions that inhibit such free combinations of digital resources, such as external regulation (Kimjeon & Davidsson, 2022), might therefore impair venture creation. Recently, increasing attention has been paid to the effect of combining digital data on venturing, for instance, its effect on venture growth (Huang et al., 2017; Schulte-Althoff et al., 2020). Digital innovation and digital entrepreneurship research have, however, predominantly focused on data in consumer-facing firms where most data that is being combined is transaction data owned by singular firms or platforms, e.g., considering financial transactions between providers and users (Huang et al., 2017) or investors and borrowers (Gomber et al., 2018), potential partners for online dating (Davidson & Vaast, 2010), ride sharers and users (Frey et al., 2019), or spectators and e-race drivers (Jarvenpaa & Standaert, 2018).

In the following, we lay out how omics data differs from other (health) data and what ventures have been created with such data. Both will inform our deeper investigation into how ventures use that data to create and grow ventures.

### Distinct characteristics of omics data for venturing

**Fig. 1:**
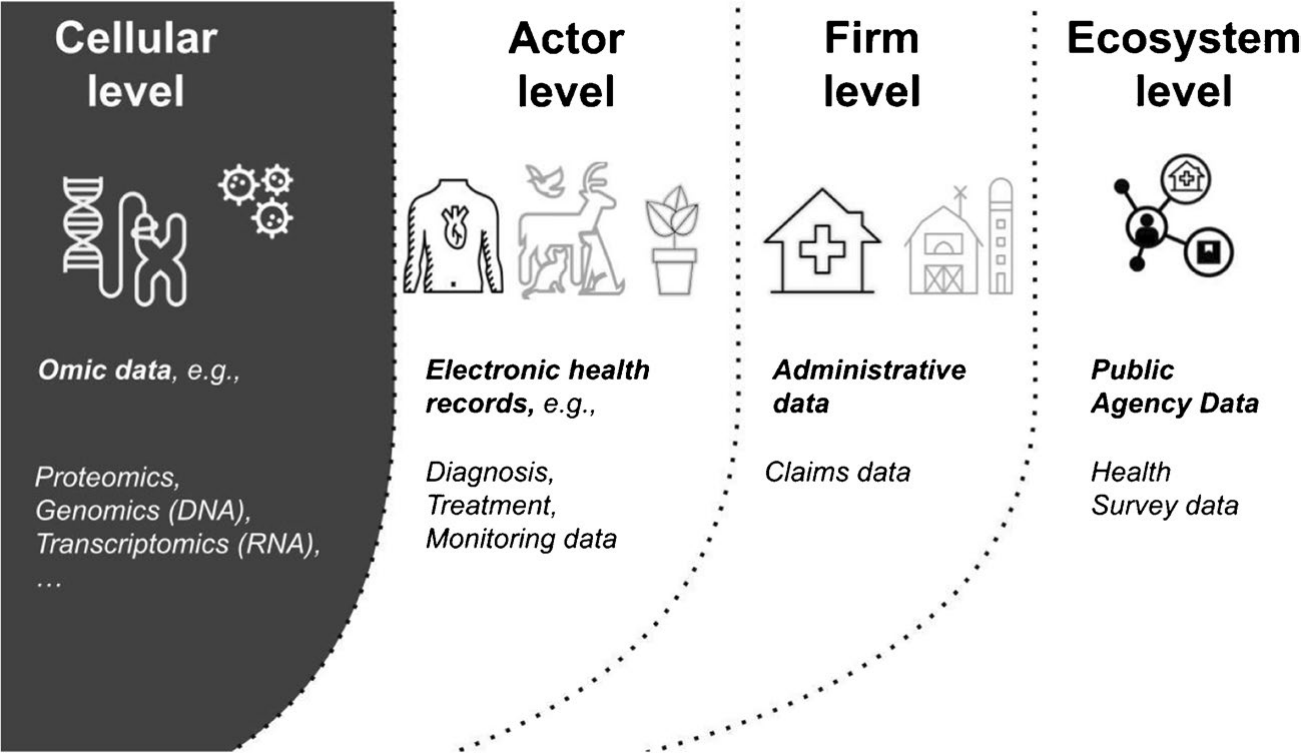
Four levels of health data

An increasing amount of health data is being produced across cellular, actor, firm, and ecosystem levels (see Fig. 1). Traditional health information systems or providers of wearable devices like Apple or Google’s Fitbit collect data of patients and healthy citizens (Gleiss et al., 2021). At the firm level, healthcare or insurance providers accumulate claims data from multiple human actors to groups in order to evaluate products and services (Bardhan et al., 2020). Health data on subordinate levels can therefore be aggregated to data on higher levels if that data follows similar structures (e.g., Kohli & Tan, 2016). Standard vocabularies for electronic health records like SNOMED or ICD-10-CM provide such structures. Here, new health data platforms become intermediaries that enforce standards and treat health data as transaction data (Fürstenau et al., 2021). Clinicians and laboratories who collect electronic health records might still diverge in how they apply data standards (Kohli & Tan, 2016), but platforms play a harmonizing role. Constantinides & Barret (2015), for instance, lay out how standards and interoperable systems over time allowed transactions of electronic health records like CT scans or X-rays which enabled data aggregation on firm and eventually ecosystem levels. This was important because such data helped inform health policy.

Advances in omics technologies have increased cost-efficiency of producing data on a *cellular level* (Manzoni et al., 2018), which is why utilizing such data has become common practice in health. Omics data, for example, from DNA is collected with sequencing technologies that translate a biological sample such as saliva or blood into bit strings. Omics data contains instructions for functions of living organisms like adaptation or natural selection and how they develop. Thereby, DNA cannot only explain alteration of biological functions but also diseases, like cancer. Moreover, this data can now be produced at scale. Generation and processing of genome sequences is now a rapid process (Shendure et al., 2017). As a result, the quantity of data has exploded since the completion of the human genome project in 2003 (e.g., Birney et al., 2017; Lander, 2011). Beyond genomics (DNA), numerous other forms of omics data can be collected, including transcriptomics (RNA), proteomics (proteins), and metabolomics (small molecules). Multiple types of omics data can be integrated to provide a more comprehensive picture of biological function at varying levels (Hasin et al., 2017). Omics technologies are based on biochemical assays that measure molecules of the same type from an organism, e.g., bacteria, animal, or human cells. Next-generation sequencing technologies, for instance, generate complete genetic or molecular profiles in high-throughput. The resolution at which such data can be captured today has thereby significantly improved. Some forms of omics data are captured at the level of a single cell, rather than representing an average of a population of cells (“Method of the Year 2013,” 2014). Prior research suggests that omics data is highly dimensional, highly regulated, and non-standardized which might affect venture creation and growth.

#### Cellular-level data are in continuous flux leading to high dimensionality

Raw omics data is regularly large in storage size (Voelkerding et al., 2009). Biological samples such as blood or saliva can be accessed through biobanks, oftentimes with tight access control (e.g., Jarvenpaa & Markus, 2018). Much omics data, however, is made publicly available by scientists who publish their data either on private repositories, digital infrastructures of their institutions, or on public digital infrastructures that both hold data and allow its free distribution (Perez-Riverol et al., 2019). Rapidly advancing scientific insights lead to constant change of data structures (Lee, 2015), however, and the broad community of scientists involved in sharing and editing omics data (Blotenberg et al., 2022) continuously adds dimensions with new methods or data being collected for ever new purposes. To sustain changes of technologies, standards, or procedures activities (Jarvenpaa & Essén, 2023), scientists capture the “data provenance” by adding further time-related metadata (Lee et al., 2017). This, again, produces even more data features. As a result, omics data becomes high-dimensional, i.e., containing a “large number of unique features or signals” (Acosta et al. 2022, p. 1773). While much health data has become high-dimensional on an individual level with image, audio, video, and historic patient data being available, omics data adds an exponentially greater number of features as the resolution of data increases (Berisha et al., 2021) and provenance keeps track of changes in scientific knowledge and technologies.

#### Cellular-level data are highly regulated

Similar to other data in healthcare, such as electronic health records (Hansen & Baroody, 2020), privacy concerns, specific consent statements, and regulation bind data to its original purpose. Omics data holds information about humans that never change over the course of a life that can hardly be anonymized and even provides information on relatives. Thus, while health data is notoriously private and highly regulated, omics data exacerbates privacy concerns (Bonomi et al., 2020). Regulations around omics data are therefore particularly strict. Ventures who seek to reuse omics data, i.e., using existing omics data for their original or for other purposes, have to navigate a considerable amount of legal red tape particularly in a legally fragmented space such as Europe where every country interprets data sharing practices, such as GDPR (General Data Protection Regulation), differently. Data access bureaucracy increases costs of data sourcing or can even make it impossible when commercial entities become excluded from usage of such personal data.

#### Cellular-level data are non-standardized

Omics data carries great potential to be used for other purposes than the ones that they have been originally created for. Reusing omics data for other purposes though relies on the ability of a data user—regularly a different person than the originator—to combine different data types from different data sources, e.g., electronic health records, psychometric data, or usage data, in order to serve these new purposes. Coverage of omics data has improved, and many new organisms have been decoded so that there are ample opportunities to combine omics data in new ways. The typical research process from which most data originates, however, has left omics data largely fragmented, non-standardized, much of it indeterminate and hence error prone (Vassilakopoulou et al., 2019). This does not necessarily refer to carelessness in the data generation step but often comes down to human error, e.g., when adding descriptive metadata or by omitting metadata altogether. Scientists produce omics data for scientific purposes (Constantiou & Kallinikos, 2015; Newell & Marabelli, 2015)—not for application in industry, let alone use by ventures. Omics data is produced to formulate and test hypotheses and publish research manuscripts (Bercovitz & Feldman, 2008; Dougherty & Dunne, 2012; George et al., 2005). As a result, such data regularly is only created for singular purposes on infrastructures that provide such data within the limits of the goals currently funded by a research project (Attwood et al., 2015). Initial steps of standardization from institutions like the GA4GH have accomplished to set some standards, for instance, on data queries, authentication, or a data use ontology, but lack widespread use because their utility still remains to be evaluated (Rehm et al., 2021) especially in clinical practice (Page et al., 2023). Like much data in the sciences (Razzak et al., 2020), omics data therefore regularly remains inconsistent and messy. This stands in the way of effective repurposing of health data, which depends on unambiguous data structures that can be similarly interpreted by originators and users of such data, especially with regard to health (Ghosh & Scott, 2011).

### Current use of omics data in ventures

Accumulation of omics data has led to an abundance of biological data that drives new areas of innovation, for example, personalized medicine (Carrasco-Ramiro et al., 2017). This has been exemplified in the recent COVID-19 pandemic where between February 2020 and August 2022 circa 6 million SARS-CoV-2 DNA sequences from 114 countries were made publicly available through the COVID-19 data portal alone. This data has been vital for producing new diagnostics, treatments, and vaccines throughout the pandemic response. To illustrate the potential of omics data for venture creation and growth, we lay out what ventures have been formed based on omics data before turning to our qualitative inquiries. We collected venture data from the start-up platform Crunchbase and provide an overview about the venture’s domains of product and services.

**Fig. 2:**
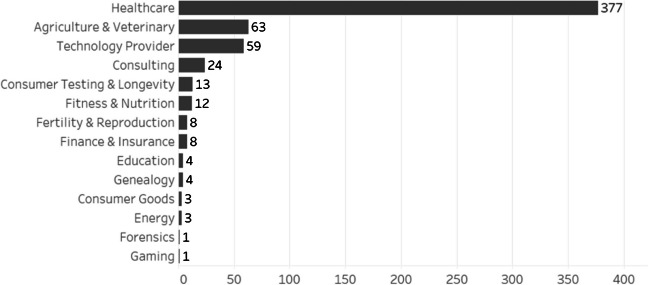
Ventures that use omics data sorted by industry (founded between 2017 and 2022)

Between 2017 and 2022, 609 new ventures have been formed that utilize omics data. Ventures use omics data across various industries, from agriculture and food to biomedical studies and healthcare. About 65% of the ventures are active within the healthcare domain (see Fig. 2), producing therapeutics via drug repurposing, drug discovery, or new diagnostic applications. China-based Abogen, for instance, uses genomic data of humans and viruses to develop cancer treatments and mRNA vaccines on the back of genomic data. For this, the venture produced and patented devices to collect and process biological samples. US firms like Immunai, Immunitas, or Vanqua Bio use omic data to model immune reactions, especially for cancer treatments. Ventures like Aviv Scientific or Minicircle seek to increase longevity of humans by assessing age-related declines using omics data, regularly as a direct-to-consumer service (see also Thiebes et al., 2020). Israeli BetterSeeds, US-based Pebble Labs, or Tu Biomics turn to omic data of plants and bacteria in soil to increase productivity of agricultural plants. Fitness ventures like UK-based Nutri-Genetix or Brazilian Progenes use omic data to suggest personalized diets for their clients. Indian Genleap combines omic data with psychometric data to provide educational advice. Table 1 provides an exemplary list of ventures who utilize omics data. Together, ventures that use omics data have accumulated investments of about $134.5 billion, the majority of which in the USA and China ($115.1b).

**Table 1:** Exemplary ventures that use omics data across industries

Venture	Industry	Service or product	Omics data
Biostrand	ICT	Life science data management and analysis platform (e.g., for microbiome or single cell analysis, population studies, or cohort stratification)	Proteomics, genomics, and other biological high-volume data of clients
Healx	Healthcare	AI platform for repurposing of existing drugs for rare diseases	Transcriptomic data is combined with phenotype data, compound and drug data, biomedical literature, and biochemistry data to produce proprietary datasets with partners, clients, and academia
BetterSeeds	Agriculture	Genome editing of crop seeds via RNA-guided DNA endonuclease activity (CRISPR)	Plant genome data, DNA/RNA sequence data of molecular tools (e.g., plasmids), producing proprietary and open omic datasets with partners
Ancestry.com	Ancestry	Prediction of genetic ethnicity for genealogy services	DNA/RNA sequences to produce a proprietary dataset of 1.4 m customer DNA samples
Abogen	Biotech	mRNA technology platform for vaccine and drug discovery, including devices to collect, preserve, and transmit biological samples for sequencing and analysis	DNA/RNA sequences to produce a proprietary dataset

In order to understand how the characteristics of omics data influence the creation and growth of ventures, we conceptualize such data as external enablers. For this, we engage in a qualitative interview study that uncovers the relevant characteristics of omics data from a digital entrepreneurship perspective.

## Methodology

This manuscript builds upon empirical insights from interviews conducted between 2018 and 2019, a focus group discussion that has been conducted at the EuroScience Open Forum 2020 (ESOF), and a series of follow-up interviews including a podcast series conducted in 2020 and 2021. We present our qualitative material in Table 2. Some interviewees preferred to remain anonymous.

**Table 2:** Interview material

	Interviewee and type	Material
1	Founder A (clinician consultancy for precision medicine), UK	Transcript (60 min, 14 p.)
2	Founder B (genome data platform for rare diseases), UK	Transcript (62 min, 16 p.)
3	Founder C (search engine for omics data and text analytics service), UK	Transcript (55 min, 18 p.)
4	Founder D (diagnostics for epigenetic data), UK	Transcript (45 min, 12 p.)
5	Founder E (plant seed database and analysis platform), Germany	Transcript (45 min, 15 p..)
6	Founder F (microbiome data platform), Germany	Transcript (60 min, 20 p.)
7	Focus group with Abel Ureta Vidal (Serial Entrepreneur and Investment Director, CMS Ventures, and Sofi Health), UK and France) Jason Mellad (CEO, Start Codon Accelerator), UK Jessica Vamathevan (Head of Strategy, European Bioinformatics Institute), UK Maria Chatzou Dunford (CEO, Lifebit), UK	Transcript (90 min, 29 p.)
8	Hans Garritzen (VP Sales and Marketing, Medisapiens), Finland	Transcript (91 min, 27 p.)
9	Maria Chatzou Dunford (CEO, Lifebit), UK	Transcript (60 min, 20 p.)
10	Abel Ureta Vidal (Serial Entrepreneur and Investment Director, CMS Ventures, and Sofi Health), UK and France	Transcript (58 min, 18 p.)
11	Lead Scientist (Dept. Agriculture, Large Biotech company), France	Transcript (58 min, 18 p.)
12	Founder G (omic data analysis platform), Netherlands	Transcript (58 min, 18 p.)
13	Founder H (cell and gene therapy), Switzerland	Transcript (56 min, 18 p.)

We selected informants with experience in entrepreneurship with omics data. They engaged in entrepreneurial activities themselves or are closely partnering with ventures. All interviewees and all participants of the focus group are regarded as opinion leaders and have worked in the data-driven life science sector for many years either on the infrastructure provider side, in industry at large multinational companies or as entrepreneurs, as well as investors of digital ventures in the life science domain. Interviews in 2018 and 2019 were held in person. Focus groups can provide deep insights into a topic of interest by laying out dissents and consensus between participants (Nili et al., 2017), enabling reflections of individual statements at individual and group levels. Thereby, focus group data can increase trustworthiness into results of a qualitative inquiry (Stahl et al., 2011). Our focus group session was held as a hybrid event with face-to-face sessions in Trieste and a virtual session. A summary was reiterated with the participants of our focus group to assure correct representation of their input at the focus group. In order to verify individual interpretations and provide us with the chance to get further details on talking points from the focus group session, we conducted follow-up interviews via video conferences. Those were partly published as podcast episodes in 2020 and 2021.

Complementary to our focus group, we interviewed founders following investigative interview techniques (Langley & Meziani, 2020) throughout which we tracked down the role of omics technologies and cellular-level data during venture creation. Interview questions (see Appendix) were informed by concepts from digital entrepreneurship literature to extend our knowledge on this subject (Urquhart et al., 2010), but formulated in ways that practitioners from the field would understand without extensive up-front explanation. We therefore conducted our interviews based on two open questions: (a) How do entrepreneurial ventures use omics data when starting their venture? (b) How do entrepreneurial ventures use omics data as they seek to grow? We produced transcripts of the entire qualitative material and engaged in open coding to collect all important topics for the research question at hand. Here, we coded the material on a sentence level on whether informants provide arguments on how data affected venture creation, including producing initial market offerings, or growth of the venture. The team of authors discussed the topics arising from the material in several group discussions. From here, the scaffolding of important data characteristics evolved. Throughout these discussions,we considered multiple mechanisms to explain how (digital) resources could be considered external resources for ventures, and we learned that most conversations spoke to a combination as the main mechanism for venturing. This was a result of our mode of inquiry, which focused on the reuse of existing omics data rather than the creation of new data by ventures. We attempted to mitigate this problem by asking follow-up questions about whether interviewees and focus group participants were creating and using their own digital resources for venturing. Subsequently, the lead author engaged in selective coding with the entire material to better describe the topics and allow for final theorizing in the full group of authors again. Table 3 provides an overview on main categories informing our propositions with exemplary quotes.

**Table 3:** Categories on how omics data affected informants during venture creation

Data categories informing propositions	Example quote
While much cellular-level data is publicly available on open platforms, this data is fitted to its original purpose	*Genetic data is easy to find on the internet. […] What really is challenging is tying that information back to individuals, revisiting one individual over the period of time or integrating different pieces of data on anyone individual. (Founder B)*
Cellular-level data that is publicly available can only be used for new purposes by experts with knowledge of its original purpose in the sciences and knowledge on the new purpose in industry	*We had a project that was scientifically really hard […] deploying it, we thought was a day, that took three months […] Companies need to be aware of their target hardware platform and if it’s a pharma company, it’s different for every single company. (Founder C)*
Ventures consider cellular-level data from public sources not reliable, independent of the purpose they intend to use it for	*You can’t rely on anything in the public, scientific data area. The formats change with every release, every half year public databases change something in their formats. This means that you have to constantly improve your interfaces. (Founder F)*
When cellular-level data is applied for new purposes, ventures need to be able to apply the data for its original purpose	*We never throw anything away, we just give it an ambiguity score of one which means it’s highly ambiguous. People normally get rid of it, but then the customer gets read of it and we cannot bring it back. (Founder C)*

## Findings

### How cellular-level data enables venture creation

#### The digital tools to collect and process cellular-level data apply to many physical objects

At several points during our interviews, it became clear that omics data is distinct from other types of health data, because the physical objects that it represents come in various forms. This produces flexibilities that ventures can leverage, especially if they are initially not fully aware of how their later product or service will look like. Sequencing techniques help collect data on entire cell samples, singular cells, or biological functions of molecules within cells, like proteins. These techniques are comparable across living beings: be it humans, animals, plants, or other samples. Founder D, for instance, laid out how their analysis pipelines would work well across physical sample types which was important because at that point they did not know what the dominant type of sample in the future will be: “we can work with any tissue- as long as its DNA […]. It does not matter if its blood, if its solid tissue, if its circulating DNA, anything, same process.”

In addition, a database of omics data can be revisited, if data privacy laws, general consent, and intellectual property laws allow. This is important for ventures because some part of omics data, especially DNA, does not change over time and remains useful over longer periods. Founder B provides genomic data on rare diseases to researchers, effectively providing a transaction platform. For him, comparing static DNA over time with changing other omics data, provided ample opportunities for the future: “we can add a huge amount of value by making this data more dynamic and interoperable. Making it easy to go back to an individual that has already been sequenced. That has a huge number of different applications whether its researchers here in London or across Europe.”

In addition, omics data is partly inherited and is thereby comparable between actors, i.e., humans, animals, or plants. This allows ancestry services to conduct their services or animal breeders to select stock animals. Founder A can track disease processes across cancer patients and families. Founder E can build a database of seeds that helps them simulate cross-breedings to optimize plant growth. For him, tools that have been developed on one domain of omics analysis can now be applied to new domains, like genomic data from microbiome living in soil or from wheat plants: “a wheat reference model was just published this year [2018] and we are working with customers who want to have this. In principle, we receive several terabytes of sequence data, then assemble them, and in the end what comes out is a refined dataset, only a few gigabytes in size.”

#### Cellular-level data is essential for initial product and service design

Interviewees and participants in the focus group unanimously underscored the necessity to frame the value propositions as science-based in that the solution to any problem these ventures are focusing on was derived from and substantiated by scientific evidence. For instance, when ventures suggest that they found a way to detect intestinal cancer earlier, existing omics data has to fit the needs of this venture in substantiating their value proposition and product claims. Concurrently, founder A clarified that “We're moving away from the intuition-based diagnosis and treatment into a much more evidence-based, big data-driven of which genetics is just one more ingredient.” Performing R&D and producing data from scratch to confirm an initial business idea or hypothesis, however, was perceived as time consuming, expensive, and hardly feasible for new ventures. Founders were able to avoid costly data collection at the beginning of their venture creation by actively using data from public sources, or in some cases through publicly funded research grants and collaborations with universities that allowed for data generation in these oftentimes high-risk projects. Focus group participants perceived the diversity of publicly available omics data important. However, utilizing such data heavily depended on a venture’s ability to link such data to smaller proprietary datasets, e.g., to group data from different studies. Maria, for instance, exemplified how they used phenotypic and genetic characteristics to support diagnostics on COVID-19.

normally the queries that you run would be like women over 40 with COVID-status positive and certain pre existing conditions and they have a certain chromosome […] all of these genetic information comes […] from hundreds of open databases and repositories out there, like ClinVar information and PubMed information. (Maria Chatzou, Lifebit)

Given the capabilities to access and potentially combine hundreds of private and public data repositories, ventures can browse omics data from millions of people, plants, and microorganisms. Freely accessible data such as ChEMBL, which offers data on “bioactive molecules with drug-like properties,” could thereby be used to train machine learning models that drive drug compound discovery, while other public data informs selection and even design of plant seeds. Sourcing omics data from these public sources saves time and money, and it offers ventures to concentrate their resources on product and service development rather than data collection. Maria, for instance, created a federated machine learning platform with her venture Lifebit that provides interpretation tools for gaining insights from public and proprietary datasets. Abel who exited Eagle Genomics in 2020—a company that generated an AI-augmented platform technology to unify, enrich, analyze, discover, and share insights from large datasets, especially for omics data on the microbiome. Founder E highlighted how they test new applications with public data, before sequencing their own proprietary data: “We use databases that are publicly available or to some short read archives with which we make comparisons or test something before we create data of this kind ourselves to see if it is possible to achieve similar results or expected results.”

Also, Jason who launched Cambridge Epigenetics before founding the incubator StartCodon highlighted how they used machine learning algorithms on publicly available genome and transcriptome data for early detection of cancer. Finally, Pfizer/BioNTech’s mRNA-based COVID-19 vaccine exemplifies the use of open data as they reference a publicly available viral sequence in the European Nucleotide Archive with the sequence number MN908947.3. Together, our informants underscored the importance of public databases for omics data in highly competitive and uncertain environments of ventures, especially in human health where the creation of data is otherwise very costly.

### Boundary conditions for omics data on venturing

#### Public data is only usable for cutting-edge scientists with industry expertise

Similar to our initial considerations, easy access to omics data seems important for its effective reuse by new ventures. Other than initially expected though, we were surprised to learn how important the process of finding such data was for venturing. For entrepreneurs, finding data for a specific purpose can be a substantial challenge, because of its high dimensionality. While the encoded DNA might only be a long bit string, consisting of A (adenine), C (cytosine), G (guanine), and T (thymine), its real value lies in combining these bit strings with other dimensions such as location, information on patients, organs, species, health, time, or sequencing technology. Our focus group participants highlighted that oftentimes they have spent considerable time searching for data of interest, because even though that data would be potentially accessible, they were note able to find it.

This is because of proprietary file formats that inhibit search and because not all metadata covering various dimensions of omics data is also easily provided. Even fundamental metadata, like time or sequencing technology, are oftentimes missing. Genomics data that is stored in proprietary data formats like low-dimensional excel spreadsheets also inhibit searching, even if they are potentially accessible to the general public on an institute’s website.

Thus, searching for omics data as well as interpreting such data requires expertise on multiple ends. As founder A put it, “The bottleneck is the management processing, analyzing and interpretation of the data. […] You need to know what to put together, how to put together and then understand the limitations of all of the different components into that particular prediction.”

Ventures, who work with omics data, therefore hire life scientists, regularly with PhDs in biology or chemistry, that are accustomed to the biological concepts, relationships between those concepts, and knowledge of potential data sources. However, these experts do not necessarily possess the digital capabilities to process such data themselves, which is why they rely on data scientists, e.g., bioinformaticians. Product managers know what clients expect and keep an eye on a tight regulatory environment.

We a three people who already have relatively deep biological knowledge, because most of our customers have that as well and we need to understand them and then we need to translate this thing for bioinformatics, so to speak, what can you make out of it. (Founder E).

For young ventures that have limited resources, but rely on experts with extensive training, this constitutes an important caveat. Ideally, new employees are very familiar with public data sources which is why ventures in this domain often evolve out of research groups or close to well-situated universities. Hans clarified that it was important for Medisapiens to be deeply integrated in an university ecosystem in Finland, for that matter, because of their “healthy combination of IT people and bioinformatics background people.” These reasons were also emphasized by Maria who valued the physical proximity of Lifebit to Cambridge University as well as to large infrastructure providers in the Cambridge region for hiring. In the podcast, Abel pointed out how Cambridge preliminary provided experts with scientific background while London provided competent product managers that were trained and nurtured in industries with highly scalable digital technologies, such as FinTech.

**Proposition 1**: While much cellular-level data is publicly available on open platforms, this data is fitted to its original purpose.

**Proposition 2**: Cellular-level data that is publicly available can only be used for new purposes by experts with knowledge of its original purpose in the sciences and knowledge on the new purpose in industry.

#### Cellular-level data in the public is considered not reliable enough to become product-ready

While it became clear throughout the interviews that public omics data was important for new ventures, founders were also cautious to use public data. Not only

You can’t rely on anything in the public, scientific data area [...] every half year public databases change something in their formats [...] you have to constantly improve your interfaces. (Founder F)

Therefore, even if sufficient omics data is accessible, the amount of work that new ventures have to put into integrating that data goes along with increasing efforts.

You can go to the [public] databases, but to relate what the reference databases have, integrate it, and present it for your patients that's where the real bottleneck is. (Founder A)

These observations were despite efforts in recent years to standardize metadata standards, such as the FAIR initiative which aims for making science data findable, accessible, interoperable, and reusable (Wilkinson et al., 2016). In principle, FAIR data allows the creation of tools and services that span across broad sets of omics data because it would take less time to integrate data from different sources. In most cases, however, informants expressed how FAIR principles were not yet fully implemented into public data infrastructures. As Maria pointed out, it is still hard to find relevant omics data in time, and it cannot be successfully sourced by a venture. The focus group discussed two major reasons for why omics data were not living up to its promises. Both reasons highlighted misalignments between the aims of individual scientists and the entrepreneurs of our focus group:

(1) FAIR principles have not yet found its way into daily practices of life scientists and other producers of omics data. When scientists submit their data, what they want to do is to comply with funder’s or a journal’s requirements for publication. “Battling with that mentality” (Jessica) is difficult for platform providers, especially when there are no well-established standards on what annotations have to be made and how these annotations are supposed to be conducted. Therefore, it is oftentimes up to the original data provider to decide what metadata they ask data producers to provide. Knowledge bases require a tremendous amount of manual curation, especially because there is a constant stream of new incoming data that is difficult to keep on top of. The COVID-19 pandemic has highlighted this as a particular bottleneck where fast turnaround from generating genomic and transcriptomic data on viruses to analysis and reporting of results is of essence. Between 2020 and 2022, the total amount of raw read sequences in the European Nucleotide Archive (ENA) grew by 20% incurring substantial resource bottlenecks (e.g., computational bottlenecks, or manual labor) which meant data availability trailed by weeks.

(2) Tools that should support adoption of FAIR principles are seldomly used outside of academia. Maria pointed out that many of these tools are regularly “homemade tools” developed by scientists within time-constrained projects for purposes limited to these projects without inbuilt sustainability. Instead of investing into existing products for data management, scientists develop tools anew with oftentimes less experienced developers, i.e., PhD students. As a result, the provided tools are not created to be used on case and do not sufficiently consider user experience, and further data is not well-annotated.

What’s the point in investing like millions on having these really expensive databases […] having all of these people annotated data and not investing like one one twentieth of that money to just getting better tools. (Maria)

Beyond the data itself, they also provide or facilitate the dissemination of tools or workflows that support the annotation of omics data that are later picked up by entrepreneurial ventures. These tools are important for balancing the need of adding more metadata while also not putting scientists off from uploading their data.

**Proposition 3:** Ventures consider cellular-level data from public sources not reliable, independent of the purpose they intend to use it for.

#### Bringing cellular-level data to new purposes requires reproducibility for its original purpose

Up until this point, we laid out how our participants of the focus group and interviewees perceived access to omics data an important obstacle for entrepreneurial ventures However, finding omics data that could be reused does not suffice for ventures. During the focus group conversation, it became clear that ventures prevail that are able to assess whether particular omics data can be used for new purposes. For Abel, “the opportunity [for science data startups] is in this ocean of data, good or bad, who is going to be the one that knows how to pick the valuable, relevant, reliable data and that you can trust.” While larger companies could potentially afford sourcing and generating omics data that is not directly related to a concrete purpose or use case, new ventures lack the resources to do so. They need to focus their human and financial resources to work for concrete purposes. Abel pointed out how important it was for his prior venture that they use “relevant data at the right time in a way that is usable for the people internally for different use cases.” It is therefore vital for ventures to quickly assess whether a particular set of omics data is applicable to a particular purpose. Jason explained that entrepreneurs turn to metadata to make these assessments. They find out, for instance, how data producers controlled for biases or technology were used to produce raw data, whether quality management systems were put in place when experiments were done, and if the data was annotated sufficiently. As Founder A pointed out with regards to public omics data, “There’s gold dust in there, but there’s a lot of noise. We know that if you perform an analysis of Mexicans, it’s going to be different from white Caucasians.”

Here, the focus group has shed light on a surprising relationship between relevance of data for application in new purposes and reproducibility of omics data for its original purposes. In other words, ventures assess how the original context of data production influences its use for the venture’s purposes.

Questions on reproducibility are regularly being in the sciences when attaining to research quality, not considering the entrepreneurial context. Maria and Jason, however, pointed out that a thorough assessment of data reproducibility is important for founders of life science ventures. Using non-reproducible or even inaccurate omics data to substantiate a product claim puts a company’s business model at high risk, because the “initial premise of their whole operation was built on a falsehood” (Jason). This is a crucial point for ventures that use omics data, because founders and investors perceive a necessity that market offerings need to be substantiated by scientific evidence. This evidence is in some cases also vetted by public bodies such as the FDA before market entry. Beyond such public institutions, private investors raise questions on the baseline data of a venture during the due diligence process in later funding rounds. Here, ventures can draw legitimacy of their data from highly reputable journals or well-known research organizations who provided that data. At the same time, however, Maria clarified that this is only one step of “ticking all the boxes” when it comes to data reproducibility for investors due diligence, which does not suffice by itself. Instead, ventures have to establish practices to quickly assess reproducibility and in some cases even conduct reproduction of data with its original purpose. Jason shared his experience as a former founder when he experienced “the pain it took after years of not having a quality management system in place of implementing it retroactively. It was a nightmare having to go back and say, oh, well, we should have been annotating all of these different bits of metadata and incorporating that into our analysis.” Spending time and resources trying to reproduce data can be detrimental for ventures. Maria discussed how her company, Lifebit, spent 6 months trying to reproduce data published by a reputable research institute. She explained that critical experimental detail needed to reproduce data was missing from the original publications. It took her team many lines of communication with individuals close to the study to gather the experimental details required to reproduce data. Maria pointed out that this approach is “not scalable” and can be hugely detrimental to start-up companies that focus on surviving at the beginning of the venturing process. Within his accelerator, Jason therefore, has put an emphasis on supporting new ventures by creating a data strategy early on. Laboratory journals can provide a good standard where every experiment is properly annotated for later use, many of those are now available in digitized form rather than handwritten notes.

When it comes to annotation these days, there’s not just the experimental details that you might need to submit. They see the details on how the instruments that were used, the settings that were on those instruments and then what you did when you were analyzing the data. So when you add all of those different components up for someone, you know like Maria, to go and actually reproduce how that data was generated, there are a lot of fields that you could potentially capture. (Jessica)

**Proposition 4:** When cellular-level data is applied for new purposes, ventures need to be able to apply the data for its original purpose. 

## Discussion

Omics technologies can serve as an external enabler for entrepreneurial ventures because it enables firms to produce health data on a cellular level. This data can be important for applications across health industries, including care or drug discovery, but also extends to applications in food and agriculture or ancestry services. Our interviews and focus groups with founders and investors exemplify how the characteristics of omics data impact the combination mechanism that affords venture creation and growth (e.g., Yoo et al., 2010; von Briel et al., 2018). We summarize our findings by showing how the characteristics of cellular-level data individually impact two activities within the combination mechanism, access and reuse, while also creating new interdependencies of both activities. We thereby situate the external enabler framework of Davidsson et al. (2020) in the context of ventures that repurpose cellular-level data. Finally, we discuss the role of ventures in repurposing cellular-level data, and why we need to rethink the timeframe at which cellular-level data becomes unsustainable, i.e., unable to serve new purposes.

**Fig. 3:**
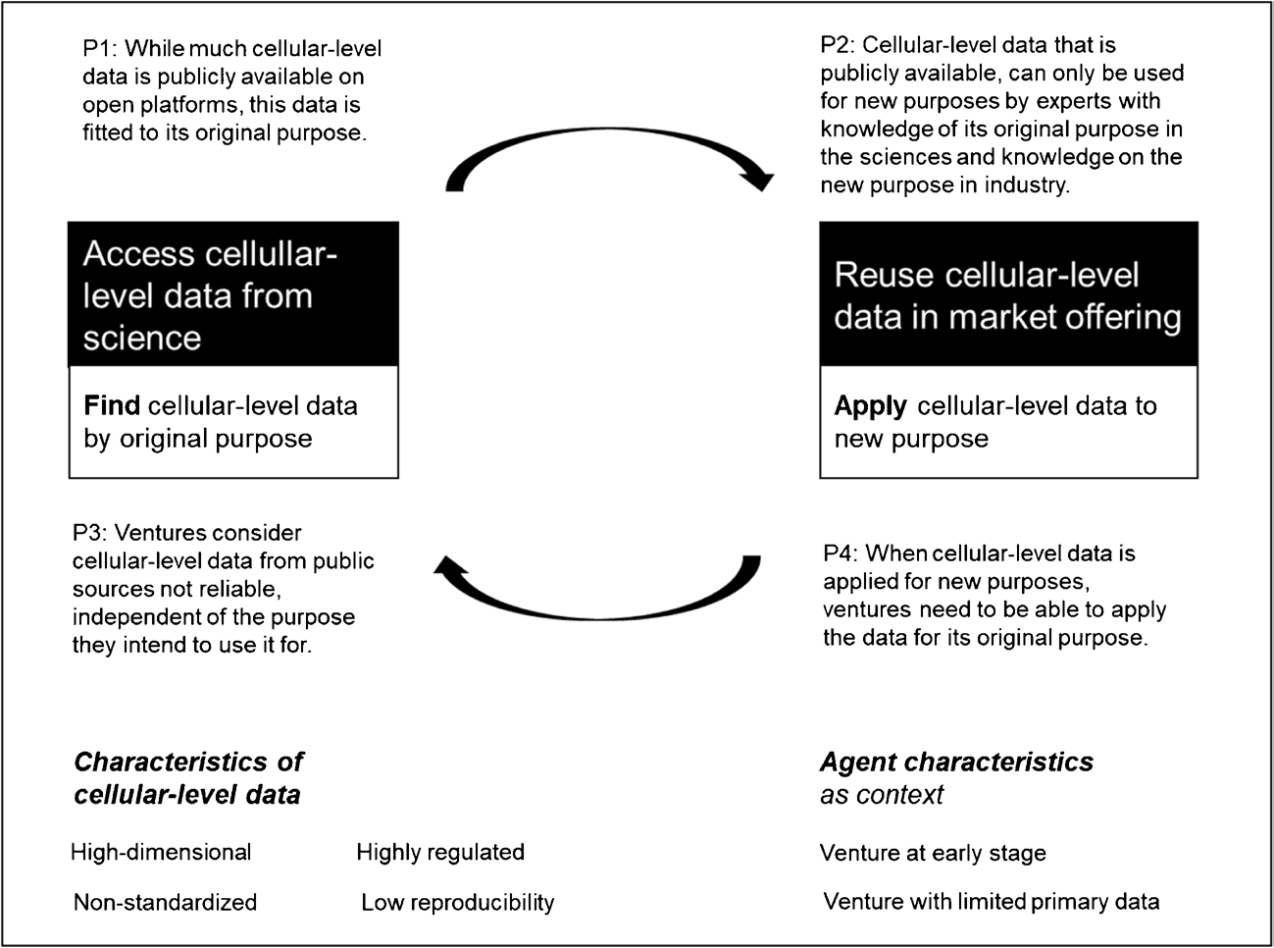
Combination mechanism enabling venturing with cellular-level data

First, we contribute to the digital innovation discourse in that we derive four propositions for how the combination mechanisms are affected by the characteristics of cellular-level data. Effortless combination of digital resources (Faulkner & Runde, 2019; Kallinikos et al., 2013) has been highlighted as a key driver for successful digital innovation. Homogeneous data, where content is successfully separated from its form (Yoo et al., 2010), is considered well-suited for creating new digital products and services (von Briel et al., 2021) because it reduces specificity and increases interoperability of tools with positive effects on venturing (von Briel et al., 2018). In this view, data is considered merely an input (Adomavicius & Tuzhilin, 2005) that can be easily accessed and then reused. Original purposes with which data was produced do not determine their applicability to new purposes in the same way as it would be for tools (Alaimo, 2022; Kallinikos et al., 2013; Yoo et al., 2010). Our empirical findings on cellular data highlight four important caveats in how lack of standards, tight regulation, a high-dimensional nature, and the need to being able to reproduce omics data for original purposes affect venturing (see Fig. 3). These dimensions substantially differ from typical empirical settings for information systems research on digital entrepreneurship or digital innovation, which tends to focus on actor-level or firm-level data, for instance, on transaction platforms (e.g., Aaltonen et al., 2021; Huang et al., 2017). In contrast to omics data, such actor-level data are predefined and standardized within company boundaries or by powerful focal actors in an ecosystem. In these contexts, data producers and data users are similar so that future purposes (e.g., Aaltonen et al., 2021; Jarvenpaa & Standaert, 2018) of data can be assessed when data is being produced. This leads to lower numbers of unique features, i.e., lower dimensionality of data, because data can be specified for this potential future purpose. However, data at the cellular level are often fragmented and indeterminate (Vassilakopoulou et al., 2019). In addition, the handling of cellular data on humans or food requires a high sensitivity to local regulations, especially with respect to privacy and data security, which extend the needs for health data to other levels (see also Bonomi et al., 2020). Among other things, there are ethical considerations that go beyond the actor level, because cellular data is not delimited to actors who provide cellular data of themselves but also includes personal information on their relatives. Our conversations underscore how successful access to cellular-level data is affected by data characteristics. Given that data is fitted to original purposes in the sciences, it requires ventures to have knowledge and the tools to assess how cellular-level data can be used for new purposes in product or service development. Given that our data largely provided insights into founders’ perceptions and expectations, further research needs to investigate the daily practices of data workers who access and reuse cellular-level data.

Second, and much to our surprise, we learned about the important role of reproducibility of omics data in the context of entrepreneurial ventures. This is important because it affects the combination mechanism as reuse and access become dually interdependent activities. Replicability, i.e., fitness of data to be applied to its original purpose, is of expectable importance for assessing the fit of data in the sciences. For this reason, tracking data provenance (Lee et al., 2017) has been proposed as an important activity to mitigate problems with changes in technology, standards, or procedures (Jarvenpaa & Essén, 2023) in the sciences. During our interviews, however, we learned how ventures also needed to assess replicability when trying to reuse cellular-level data for new purposes. This is why, we theorize that repurposing affords the need to access more cellular-level data because replicability needs to be tested and changes to data sources might need to be made more often when data is considered unfit for reuse. Focus group participants highlighted how they evaluate how the original data collection influences their reuse for new purposes. In a knowledge base that offers aggregated data, ventures rely on the accuracy of data, for instance, that a biomarker for a particular disease is valid, e.g., a signal in the genetic code of a virus or a human cell. If a venture sources cellular data from an archive, it needs to be able to link said data with other cellular data. Ventures thereby rely on data producers to provide correct metadata, such as sequencing information. Here, ventures need to be aware of underlying biases in cellular data, e.g., such as sourcing biases in large genome initiatives (Freeman et al., 2020), that can be hard to assess because of the high-dimensional character of cellular data. Only when ventures engage in producing data practices such as dimensional reduction to high-dimensional, they are able to assess their value for repurposing. Ventures assume that if data cannot be applied reliably for their original purpose, they would also not be applicable to other purposes. This is especially important for healthcare ventures, whose claims are scrutinized not only by customers and investors, but also by government agencies for the scientific basis of their claims of no harm to patients.

Third, our conversations revealed that sourcing omics data from publicly available infrastructures in the life sciences has an important impact on venture creation in the health domain as sciences produce new knowledge about cellular-level mechanisms and novel technologies to create and manipulate data. As laid out in Table 3, omics data are considered (a) fit-for-purpose and (b) not reliable. Founders oftentimes associate these data characteristics with their origin in the sciences, which largely complements existing conceptual remarks on innovation with data (Ghosh & Scott, 2011; Razzak et al., 2020). We thereby learned that the relationship between access to omics data and its successful use in a product during venture creation is mediated by a venture’s ability to engage in activities of repurposing; i.e., ventures use data that was originally created for purposes within basic science for new purposes related to developing a product or service. Thus, the question on whether data can be used for future—at the time of creation unknown—problems (e.g., Alaimo, 2022; Yoo et al., 2010) has to be resolved by the digital venture. Depending on whether a venture succeeds in combining omics data and digital tools for a new product or service affects its ability to enter and sustain in the market. This is an important finding because it underscores the need for new ventures to assess the original purpose for which the data was created, the impact on the data’s characteristics, and the need for a venture to develop means to modify the data for a new purpose. The additional investment associated with such activities contrasts with the assumption that providing public cell-level data, for instance, through open data initiatives to large public organizations, affords the creation of ventures. Instead, we learn that the problem of whether omics data can be brought to new purposes is currently resolved by organizations with notoriously small funding–entrepreneurial ventures. While ventures might not need to invest into creating primary data, they need to setup processes and tools to amend secondary data to their purpose. It would be up to future research, to assess whether and to what extent these additional investments outweigh the utility of producing primary data tuned to a specific service or product. In the case of omics data, the nature of the investment-heavy technology, its scientific context, and the highly regulated, often clinical environment seem to make the use of secondary data at the cellular level the main option for new ventures.

Finally, our observation that ventures who work with cellular-level data spend considerable resources on repurposing data introduces a new actor into the discourse on sustainability of data. This seems an important observation, because it questions the time spans at which data becomes unusable for repurposing and it asks who is involved in making data sustainable. Extant research remarks that data becomes unusable and therefore unsustainable in a “distant future – that is, time across technological and human generations” (Jarvenpaa & Essén, 2023). We have learned in our study that ventures are solving problems of unsustainable data at the cellular level, collected only months or sometimes years ago. Although the life sciences have been studying mechanisms at the cellular level for more than a century—the first Nobel Prize in genetics was awarded to Albrecht Kossel (1910)—the life sciences are still making great strides in this area. Most omics technologies are only up to two decades old. Massive parallel sequencing, for example, has only been available to the public since 2005. Even within the same technological and human generations, omics data seemingly becomes hard to use for new purposes (Blotenberg et al., 2022). Ventures operate on a much different time scale then large public data providers, because of their need to quickly create marketable offerings and grow. This underscores a temporal mismatch between actors and a data sustainability process that so far has been considered to span over long times. Further empirical research could shed light on how quickly data becomes unsustainable at the cellular level and what practices companies use to reuse data.

To the best of our knowledge, vetting the potential of data being reused for different purposes as well as the practices in how this is being accomplished (e.g., Alaimo, 2022) is new to the digital entrepreneurship discourse. Vetting this potential might be particularly important for ventures in heavily regulated environments such as healthcare, because we know that fluid regulations might have a detrimental effect on the performance of the combination mechanism (Kimjeon & Davidsson, 2022). At the same time, this finding might also generalize to other ventures that heavily rely on omics data. We would therefore urge further research to study the direct effects of these characteristics on the development of marketing offerings during venture creation and growth.

This study on venturing and cellular data is not without limitations. Our result might be affected by the inherent focus on omics technologies producing cellular data, particularly on genomics and proteomics. While we covered a broad range of different applications in our sampling, other technologies like mass spectrometry might have differing effects on venturing because they might produce varied data characteristics. We further lay out how the rapid development of omics technologies affects venturing. It should be noted that many technologies have undergone periods of rapid change, initially and before revolving around a dominant design. We cannot rule out that the effects of cellular data on venturing will change over time. By conducting our interviews over extended periods of time, we tried to mitigate short-term effects of technology development and standardization attempts.

### Supplementary information

ESM 1 (DOCX 207 kb)

## Data Availability

Qualitative data from selected interviews and the focus group are publicly available on the podcast series https://www.podomatic.com/podcasts/dataforlife/episodes/2020-10-15T01_21_06-07_00). Further qualitative interview data supporting the findings of this study are available on request from the corresponding author. Some interview data are not publicly available due to restrictions that could compromise privacy of research participants or because of proprietary information on the companies involved.
