# Supplementary material for: Digital entrepreneurship from cellular data: How omics afford the emergence of a new wave of digital ventures in health
Source: Electron Mark. 2023 Sep 16;33(1):48. doi: 10.1007/s12525-023-00669-w (PMC10505108; doi:10.1007/s12525-023-00669-w)
Supplement: Supplementary file 1 — (DOCX 207 kb) [file 12525_2023_669_MOESM1_ESM.docx]

# Appendix

**Interviews (2018-2020)**

1. Introduce me to [Company name] and tell me something about you
   1. Number employees / amount of funding / founding year
   2. Current role & responsibilities
   3. What is your current business model?
   4. Main source of revenue
   5. Core customers (B2B/B2C, Institution)
   6. Core channel (Software/Service per unit)
2. What kind of data are you working with?
   1. What role does omics data play in your venture?
   2. How is it collected and processed?
3. Who are your main partners and what capabilities do they provide?
   1. Main partner types
   2. Relationship type (contract, on-equity, informal …)
4. What data is provided by others? (raw data, aggregated models)
   1. How is this data used?
   2. Are third parties involved in data collection, preparation or analysis (i.e., algorithms, development)
5. What is the output you provide to your clients and how specific is it?
   1. How diverse are the fields of application for your product? (across locations, industries …)
   2. How specific or how generic is your output for clients?
6. How do you position your offering in the market?
   1. What role does IP play for your business?
7. What are your core capabilities as of today?

**Focus group (2020) on boundary conditions for in digital entrepreneurship with omics data**

1. Which role do third parties, including government, investors, universities, and the broader private sector, play in the co-evolution between digital technology change and entrepreneurship?
2. What is the role of location for engaging in digital entrepreneurship with omics data?
3. How does the government/policies (e.g. IP) impact innovation and entrepreneurship with omics data?
4. How does having a secure intellectual property environment or a lack of it impact innovation and entrepreneurship?
5. How do different economies/cultures/heritage compare in terms of innovation/entrepreneurship?
6. What is the role of innovation and entrepreneurship in low versus technology industries?

**Follow-up interviews (2021-2022)**

1. Who are you and what is your current role?
2. Give us a brief overview of your career path. Where did you receive your education and where did you train before starting your company?
3. Tell us about your company (how many people do you employ/amount of funding, founding year).
4. What is your main product or service?
   1. What is your main source of revenue and who are your main customers?
   2. Who are your main partners? What is their main input to your offering?
   3. Tell us about competition in your sector.
5. Why did you start a company?
6. What kind of data are you working with? How is it collected and processed? By whom?
   1. What role do omics data play for creation of your venture?
   2. How kind of omics data do you use?
   3. How do you incorporate omics data?
   4. What role does openness of omics data play for your offering?
7. What role did location / your ecosystem play for creation of your venture?
